# Genetic architecture of resistance to plant secondary metabolites in *Photorhabdus* entomopathogenic bacteria

**DOI:** 10.1186/s12864-025-12067-x

**Published:** 2025-10-30

**Authors:** Anja Boss, Stefan Toepfer, Matthias Erb, Ricardo A. R. Machado

**Affiliations:** 1 Experimental Biology, Institute of Biology, University of Neuchâtel, CH-2000 Neuchâtel, Switzerland; 2 CABI, CH-2800 Delémont, Switzerland; 3 Institute of Plant Sciences, University of Bern, CH-3013 Bern, Switzerland

**Keywords:** Experimental evolution, Targeted engineering, Benzoxazinoid resistance, Cross-resistance, Collateral sensitivity, Entomopathogenic nematodes, Biocontrol agents, Agricultural pests, Sustainable agriculture

## Abstract

**Background:**

Entomopathogenic nematodes of the genus *Heterorhabditis* establish a symbiotic association with *Photorhabdus* bacteria. Together, they colonize and rapidly kill insects, making them important biological control agents against agricultural pests. Improving their biocontrol traits by engineering resistance to plant secondary metabolites (benzoxazinoids) in *Photorhabdus* symbiotic bacteria through experimental evolution has been shown to increase their lethality towards benzoxazinoid-defended larvae of the western corn rootworm, a serious crop pest of maize, and it is therefore a promising approach to develop more efficient biocontrol agents to manage this pest. To enhance our understanding of the genetic bases of benzoxazinoid resistance in *Photorhabdus* bacteria, we conducted an experimental evolution experiment with a phylogenetically diverse collection of *Photorhabdus* strains from different geographic origins. We cultured 27 different strains in medium containing 6-methoxy-2-benzoxazolinone (MBOA), a highly active benzoxazinoid breakdown product, for 35 24 h-cycles to select for benzoxazinoid-resistant strains. Then, we carried out genome-wide sequence comparisons to uncover the genetic alterations associated with benzoxazinoid resistance. Lastly, we evaluated the resistance of the newly isolated resistant *Photorhabdus* strains to eight additional bioactive compounds, including 2-benzoxazolinone (BOA), nicotine, caffeine, 6-chloroacetyl-2-benzoxazolinone (CABOA), digitoxin, fenitrothion, ampicillin, and kanamycin.

**Results:**

We found that benzoxazinoid resistance evolves rapidly in *Photorhabdus* in a strain-specific manner. Across the different *Photorhabdus* strains, a total of nineteen nonsynonymous point mutations, two stop codon gains, and one frameshift were associated with higher benzoxazinoid resistance. The different genetic alterations were polygenic and occurred in genes coding for the EnvZ/OmpR two-component regulatory system, the different subunits of the DNA-directed RNA polymerase, and the AcrABZ-TolC multidrug efflux pump. Apart from increasing MBOA resistance, the different mutations were also associated with cross-resistance to 2-benzoxazolinone (BOA), nicotine, caffeine, and 6-chloroacetyl-2-benzoxazolinone (CABOA) and with collateral sensitivity to fenitrothion, ampicillin, and kanamycin. Targeted mutagenesis will provide a deeper mechanistic understanding, including the relative contribution of the different mutation types.

**Conclusions:**

Our study reveals several genomic features that are associated with resistance to xenobiotics in this important group of biological control agents and enhances the availability of molecular tools to develop better biological control agents, which is essential for more sustainable and ecologically friendly agricultural practices.

**Supplementary Information:**

The online version contains supplementary material available at 10.1186/s12864-025-12067-x.

## Background

Bacterial symbionts provide important services and influence different life history traits of their hosts. They can provide or assist their host in the acquisition of essential nutrients, aid in the digestion and detoxification of food components, increase pathogen and parasite resistance, improve general stress tolerance, and mediate host colonization [1–10]. Given the wide variety of services that bacterial symbionts provide to their host, it is often proposed that bacterial symbionts could be engineered to enhance host performance [7, 11–14]. In this context, it has recently been discovered that the lethality of entomopathogenic nematodes against the larvae of the western rootworm (WCR), *Diabrotica virgifera virgifera* LeConte 1868, can be increased by engineering benzoxazinoid resistance in their bacterial symbiotic partners [15]. This provides a good example of the feasibility of enhancing host performance by engineering symbiotic traits [15].

Entomopathogenic nematodes are insect-feeding, soil-dwelling threadworms that live in symbiosis with insect-pathogenic bacteria [16–18]. There are two main multispecies entomopathogenic nemato-bacterial complexes. One composed of *Steinernema* nematodes that establish symbiosis with *Xenorhabdus* bacteria, and the second one composed of *Heterorhabditis* nematodes that establish symbiosis with *Photorhabdus* bacteria [19–22]. In both cases, the nematodes colonize soil-borne insects and release their symbiotic bacteria [23]. The bacteria then pre-digest and kill the insect by releasing insecticidal secondary metabolites, lytic enzymes, and immune suppressors [24–29]. The nematodes feed on the liquefied insect tissues, reproduce, reestablish symbiosis with the bacteria, and abandon the exploited cadaver in search of a new host [17]. Given their rapid insect-killing abilities, these organisms are used as biological agents against agricultural pests.

Normally, insects are very susceptible to the attack of entomopathogenic nematodes [30–33]. However, certain insects have evolved sophisticated mechanisms to survive their attack, as is the case of WCR [34]. These insects can selectively accumulate a variety of plant secondary metabolites, including two classes of benzoxazinoids, benzoxazolinones and benzoxazinones, and use them against natural enemies, including entomopathogenic nematodes and their symbiotic bacterial partners [15, 34–36]. Hence, WCR larvae are partially resistant to the attack of non-adapted *Heterorhabditis* entomopathogenic nematodes [15, 34, 36]. The increased resistance is associated with two different mechanisms. On the one hand, the WCR larvae release the benzoxazolinone 6-methoxy-2-benzoxazolinone N-glucoside (MBOA-Glc), which repels the nematodes. On the other hand, WCR larvae accumulate the benzoxazinone 2-hydroxy-4,7-dimethoxy-1,4-benzoxazin-3-one O-glucoside (HDMBOA-Glc) and release its breakdown product the benzoxazolinone 6-methoxy-2-benzoxazolinone (MBOA) upon nematode attack, both of which are directly toxic to the *Heterorhabditis* nematodes and their *Photorhabdus* bacterial symbionts [15, 34, 36].

Previous studies have shown however that not all *Heterorhabdits* populations and *Photorhabdus* strains are susceptible to the BXD-mediated defensive mechanisms of the WCR, as some of them have evolved resistance to benzoxazinoids [15, 31, 36]. Nematode populations present in North America that have a longer co-occurrence history with WCR are overall more lethal than European nematode populations that have no history of interactions with these insects [36]. In addition, through experimental evolution, both nematodes and symbiotic bacteria can rapidly evolve benzoxazinoid resistance under laboratory settings [15, 36]. Breeding a benzoxazinoid-susceptible nematode isolate in WCR larvae for five generations resulted in higher behavioural and metabolic resistance and superior benzoxazinoid-dependent lethality towards WCR larvae [36]. Similarly, culturing *Photorhabdus* strains in BXD-containing medium for a few cycles resulted in the evolution of BXD resistance [15]. Using targeted genetic engineering, the aquaporin-like channel gene aqpZ was identified as a BXD-resistance factor [15]. Transferring the BXD-resistant strains to their original nematode hosts increases the ability of the symbiotic pair to kill WCR larvae [15]. These studies encourage further efforts to gather a deeper understanding of the genetics of benzoxazinoid resistance in this symbiotic pair [37].

In this study, we focused on understanding the molecular mechanisms that underlie benzoxazinoid resistance in *Photorhabdus* and on investigating the consequences of evolving BXD resistance in the context of collateral sensitivity and cross-resistance to other environmental xenobiotics and growth abnormalities. To this end, we carried out experimental evolution in a collection of 27 *Photorhabdus* strains representing seven taxa and twelve countries of origin, carried out genome-wide genetic characterizations, and profiled bacterial resistance and growth under different culturing conditions. Our study uncovers the polygenic nature of benzoxazinoid resistance and provides additional evidence for the feasibility of improving biocontrol-relevant traits efficiently in entomopathogenic bacteria.

## Materials and Methods

### Bacterial strains

*Photorhabdus* strains used in this study have been isolated from different *Heterorhabditis* nematodes as described in previous literature [36, 38–41] (Table 1). To this end, *Galleria mellonella* larvae were infested with one hundred infective juveniles. Two to three days later, insects were dissected with a sharp blade, and their bacteria-digested internal organs were spread onto LB agar plates. Pure cultures were obtained by replating *Photorhabdus*-like colonies. Bioluminescence production was used to confirm successful symbiont isolations. Isolated bacteria were molecularly identified using 16S rRNA gene sequences and whole genome sequences [18, 42]. Whole genome sequencing procedures are detailed below. Bacterial strains were regularly cultured and maintained in LB plates at 28 °C [43].

**Table 1:** Bacterial strains used in the current study. Strain IDs were used to refer to the different bacterial strains throughout the manuscript. Scientific species names, strains, species of the nematode hosts, and country of origin are indicated

Strain ID	Bacterial species/strain	Nematode host	Origin
EN01	*P. laumondii* subsp. *laumondii* EN01-24	*H. bacteriophora*	Germany
DE2	*P. laumondii* subsp. *laumondii* DE2-34	*H. bacteriophora*	
DE6	*P. laumondii* subsp. *laumondii* DE6-41	*H. bacteriophora*	
A	*P. laumondii* subsp. *laumondii* A-59	*H. bacteriophora*	
DIA	*P. laumondii* subsp. *laumondii* DIA-62	*H. bacteriophora*	
PT1	*P. thracensis* PT1-31	*H. bacteriophora*	Portugal
IT6	*P. laumondii* subsp. *laumondii* IT6-44	*H. bacteriophora*	Italy
IR2	*P. kayaii* IR2-43	*H. bacteriophora*	Irland
HU2	*P. kayaii* HUG-39	*H. bacteriophora*	Hungary
0943	*P. thracensis* 0943–58	*H. bacteriophora*	Turkey
MG6286	*P. laumondii* subsp.* laumondii* MG6286-45	*H. bacteriophora*	Switzerland
TT01	*P. laumondii* subsp. *laumondii* TT01^T^	*H. bacteriophora*	Trinidad and Tobago
B	*P. kleinii* B-49	*H. bacteriophora*	United States
S5P8	*P. laumondii* subsp. *laumondii* S5P8-50	*H. bacteriophora*	
S7	*P. laumondii* subsp. *laumondii* S7-51	*H. bacteriophora*	
S8	*P. kleinii *S8-52	*H. georgiana*	
S9	*P. kleinii* S9-53	*H. georgiana*	
S10	*P. kleinii* S10-54	*H. georgiana*	
S12	*P. laumondii* subsp. *laumondii* S12-55	*H. bacteriophora*	
S14	*P. laumondii* subsp. *laumondii* S14-60	*H. bacteriophora*	
S15	*P. laumondii* subsp. *laumondii* S15-56	*H. bacteriophora*	
KC	*P. laumondii* subsp. *laumondii* KC-57	*H. bacteriophora*	
BIO	*P. laumondii* subsp. *laumondii* BIO-48	*H. bacteriophora*	
MEX20	*P. khanii* subsp. *guanajuatensis* MEX20-17^ T^	*H. atacamensis*	Mexico
CN4	*P. bodei* CN4-25	*H. beicherriana*	China
LJ	*P. bodei* LJ24-63^ T^	*H. beicherriana*	
IL9	*P. laumondii* subsp. *laumondii* IL9-35	*H. bacteriophora*	Australia

### Experimental evolution

Experimental evolution was carried out as described previously [15]. To this end, the different bacterial strains were cultured in LB broth containing 6-methoxy-2-benzoxazolinone (MBOA)(Sigma-Aldrich, Switzerland) at a concentration of 200 μg/ml (MBOA-selected strains). Controls were cultured in MBOA-free LB medium (LB-selected strains). Liquid cultures were incubated at 28 °C with constant shaking (180 rpm) for 24 h. After this time period, one millilitre of the resulting bacterial cultures was transferred to 30 ml of fresh LB medium, with or without MBOA. This procedure was repeated for a total of 35 culturing cycles. After the last culturing cycle, 50 μl of MBOA-selected cultures and 50 μl of LB-selected control cultures were individually plated on LB agar plates. Three single colonies of each strain were selected, sub-cultured individually on LB agar plates (3 days at 28 °C), and then in liquid LB (16 h at 28 °C and constant shaking at 180 rpm), and preserved as glycerol stocks (40% v/v at −80 °C). The MBOA-selected and LB-selected strains were used to evaluate their resistance to different metabolites as described below (Table 2). In addition, the ancestral, MBOA-selected, and LB-selected strains were subject to whole genome sequencing and variant calling analysis to assess the genetic changes suffered during the MBOA selection procedure. Strains DE2, DE6, HU2, MG6286, BIO, CN4, and LJ did not recover from the glycerol stocks and could not be genotyped.

**Table 2:** Concentration ranges, Chemical Abstracts Service (CAS) numbers, and biological activities of the different metabolites used in this study. All metabolites were purchased in Sigma-Aldrich

Chemical	CAS number	Concentration range	Biological function
2-benzoxazolinone (BOA)	59–49-4	0—1000 µg/ml	Insecticidal benzoxazinoid produced by Poaceae
Nicotine	54–11-5	0–6.25 µl/ml	Alkaloid produced by *Nicotiana* spp. plants and sequestered by different insects incl. *Manduca sexta*
Caffeine	58–08-2	0—2000 µg/ml	Insecticidal alkaloid
6-chloroacetyl-2-benzoxazolinone (CABOA)	54,903–10-5	0—40 µg/ml	Inhibitor of germination and radicle development
Digitoxin	71–63-6	0—1600 µg/ml	Cardiac glycoside produced by the genus of *Digitalis* with insecticidal properties
Fenitrothion	122–14-5	0—5 µl/ml	Phosphorothioate (organophosphate) insecticide
Ampicillin	69–52-3	0—100 µg/ml	Penicillin antibiotic
Kanamycin	25,389–94-0	0–12.5 µg/ml	Aminoglycoside antibiotic
6-methoxy-2-benzoxazolinone (MBOA)	591–30-0	0–400 µg/ml	Insecticidal, bactericidal, and nematocidal benzoxazinoid produced by Poaceae

### Whole-genome sequencing and variant calling analysis

Bacterial genomic DNA was extracted using the GenElute Bacterial Genomic DNA Kit (Sigma-Aldrich, United States) following the manufacturer’s instructions. Genomic libraries were prepared using the TruSeq DNA PCR-Free LT Library Prep kit (FC-121–3003), and indexed libraries were pooled at equimolar concentrations and sequenced (2 × 150 bp) on an Illumina HiSeq 3000 instrument. Raw Illumina reads were quality trimmed by using Trimmomatic 0.36 (options: SLIDINGWINDOW:4:8 MINLEN:127) [44]. Resulting reads were assembled using SPAdes 3.10.1 (k-mer sizes of 21, 33, 55, 77, 99 and 127 bp), and the obtained contigs were assembled to scaffolds using SSPACE 3.0 with default options [45]. Gaps were filled using GapFiller 1.10 [46]. Scaffolds with a mean read depth of less than 20% of the median read depth for the longer scaffolds (≥ 5,000 bp) and scaffolds shorter than 200 bp were removed. The final assemblies were polished using Pilon 1.22 [47]. To determine the genetic alterations associated with the observed changes in benzoxazinoid resistance following experimental evolution, a subset of MBOA-selected and LB-selected strains was selected and their genomic differences were evaluated pair-wise by variant calling analyses using Snippy 4.6.0 (Victorian Bioinformatic Consortium, Australia, https://github.com/tseemann/snippy). Gene sequences of ancestral, LB-, and MBOA-selected strains are available as a supplementary material (Data S1). Protein structure homology modelling was conducted using the SWISS-MODEL server [48].

### Evaluation of resistance to the different metabolites in vitro

To evaluate the resistance of the MBOA-selected strains and the LB-selected control strains to different xenobiotics, we measure bacterial growth in LB medium with or without the different metabolites at different concentrations in 384-well microtiter plates (Greiner Bio-One, Austria) following the protocols described previously [15, 49]. Briefly, bacterial cultures grown for 16 h at 28 °C in LB (25 g/l, Carl Roth, Switzerland) were diluted to an OD_600_ = 0.05 and ten microliters of the resulting bacterial solutions were inoculated into 70 μl of LB liquid medium (12.5 g/l) containing one of the following metabolites: 2-benzoxazolinone (BOA), nicotine, caffeine, 6-chloroacetyl-2-benzoxazolinone (CABOA), digitoxin, fenitrothion, ampicillin, kanamycin, or 6-methoxy-2-benzoxazolinone (MBOA). These metabolites were selected due to their potential of being encountered by *Photorhabdus* bacteria as some of them are accumulated by insects after feeding on plants (nicotine, caffeine, digitoxin), are produced or exuded by plants (BOA, CABOA) or insects (MBOA), are common pesticides (fenitrothion) or antibiotics (ampicillin, kanamycin) that can accumulate in soils [34–36, 50–57]. Different concentrations of each metabolite were used as indicated in Table 2. Pilot experiments were used to establish the concentration range in which bacterial growth was unaffected at the lowest concentrations and fully suppressed at the highest concentrations. Bacterial growth was estimated by measuring optical densities at 600 nm at regular intervals for a period of 48 h using a Synergy™ HT Multi-Detection Microplate Reader (BioTek, United States). Plates were maintained at 28 °C and gently shaken orbitally before each measurement.

### Statistical analysis

All statistical tests were carried out using Sigma Plot 15.1.1.16. Growth inhibition 50 (GI_50_) datasets were statistically assessed by two-way ANOVA. Bacterial growth was statistically assessed by two-way repeated measurements ANOVA. Holm post hoc tests were used for multiple comparisons. Normality and equality of variance were verified using Shapiro–Wilk test.

## Results

### Rapid evolution of benzoxazinoid resistance is strain-specific

**Fig. 1:**
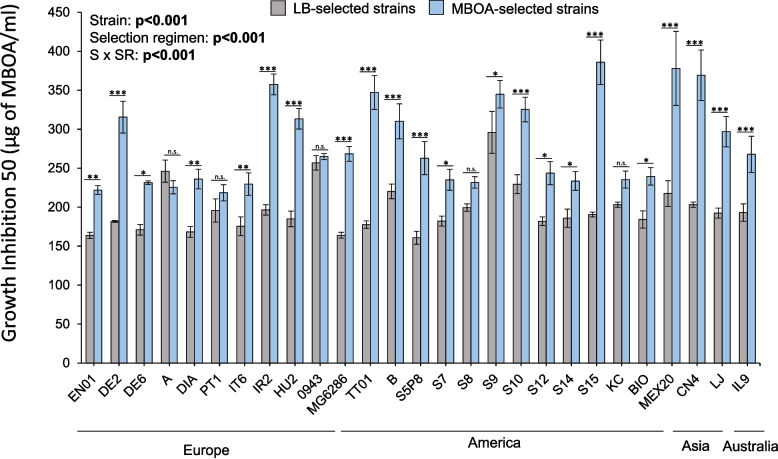
Experimental evolution in MBOA-containing culture medium results in an increased MBOA resistance in a strain-specific manner. Mean (± SEM) MBOA concentrations that inhibit bacterial growth by 50% (Growth Inhibition 50, GI_50_) in the bacterial strains selected in LB-medium (LB-selected, grey bars) and in the bacterial strains selected in MBOA-containing medium (MBOA-selected, blue bars). Bacterial strains are grouped by their geographic origin. Asterisks indicate significant differences in GI_50_ values between LB-selected and MBOA-selected strains by two-way ANOVA with Holm’s multiple-comparisons tests (*: p < 0.05, **: p < 0.01, ***: p < 0.001). Experiments were conducted four to six independent times with one replicate each time (n = 4–6). MBOA: 6-methoxy-2-benzoxazolinone

Twenty-seven phylogenetically diverse *Photorhabdus* strains whose *Heterorhabditis* nematode hosts were collected at different geographical places were used for experimental evolution in 6-methoxy-2-benzoxazolinone (MBOA) containing LB medium (Table 1). After 35 culturing cycles, significant differences in MBOA resistance levels between MBOA-selected and LB-selected strains were observed in a strain-dependent manner, being often higher in the MBOA-selected strains (Fig. 1). MBOA resistance, estimated as the MBOA concentration that reduces bacterial growth by 50% (GI_50_), ranged from 166 to 385 µg of MBOA/ml across all the different MBOA-selected and LB-selected strains (Fig. 1). MBOA resistance was increased by about two folds in strains TT01, IR2, S15, and CN4. MBOA resistance was increased by about one-fold in strains DE2, HU2, MG6286, S5P8, MEX20, and LJ. MBOA resistance was increased by 16–42% for most of the remaining strains. No significant changes in MBOA resistance were observed for strains A, PT1, 0943, S8, and KC (Fig. 1). Taken together, MBOA resistance can rapidly evolve in laboratory settings in *Photorhabdus* bacteria.

### Highly polygenic architecture underlies benzoxazinoid resistance in Photorhabdus

**Fig. 2:**
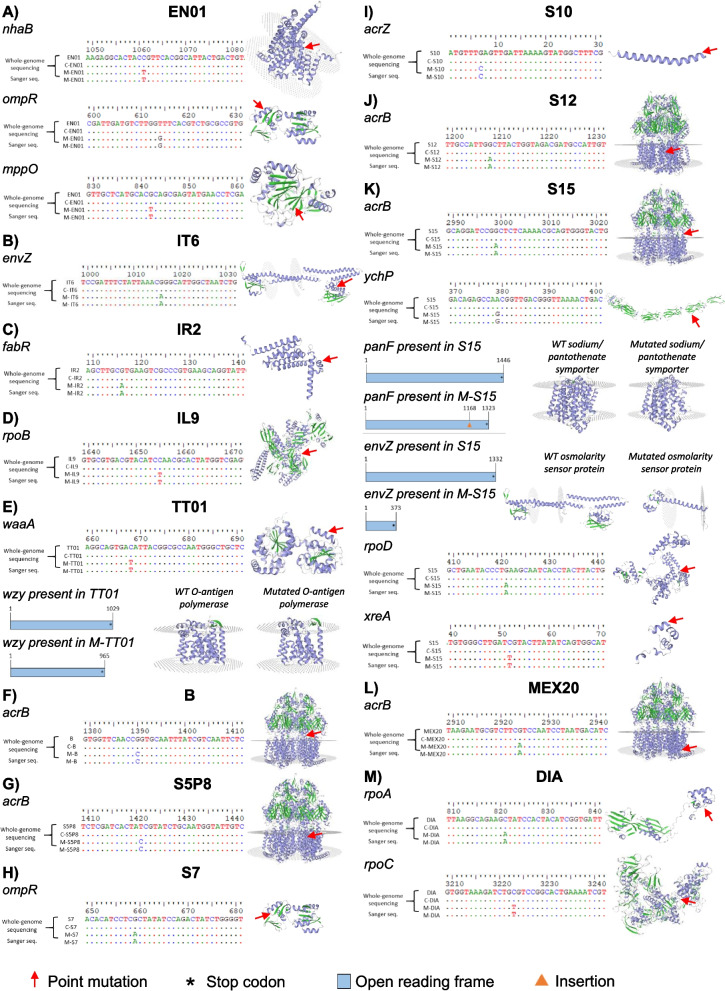
Experimental evolution in MBOA-containing culture medium results in multiple genetic mutations. Nonsynonymous gene mutations found in MBOA-selected strains (M-) compared to LB-selected strains (C-) and ancestral strains determined by variant calling analyses and Sanger sequencing are shown. Mutations occurring in: A) M-EN01, B) M-IT6, C) M-IR2, D) M-IL9, E) M-TT01, F) M-B, G) M-S5P8, H) M-S7, I) M-S10, J) M-S12, K) M-S15, L) M-MEX20, and M) M-DIA. Blue schemes represent open reading frames. Asterisks represent stop codons. If the protein structure does not change, only the model of the ancestral strain is shown, including a red arrow pointing at the place of a single nucleotide polymorphism (SNP). If the protein structure differs, protein structure models for the ancestral and MBOA-selected strains are shown. MBOA: 6-methoxy-2-benzoxazolinone

Genome-wide comparisons between ancestral, MBOA-selected, and LB-selected strains revealed that multiple genes are associated with MBOA resistance in *Photorhabdus* (Fig. 2, Table 3). Across all the genetic comparisons, a total of nineteen nonsynonymous point mutations, two stop codon gains, and one frameshift, occurring exclusively in the MBOA-selected, but not in the LB-selected strains, were identified (Fig. 2, Table 3, Data S1). These mutations are predicted to impact different cellular processes, including transmembrane transport, oxidoreductase activity, osmoregulation, membrane fatty acid, lipopolysaccharide and o-antigen biosynthesis, and DNA transcription (Table 3). Although the mutations associated with MBOA resistance occur in a strain-specific manner, several common elements were observed, but no identical mutations were observed in any of the tested strains. In particular, genetic alterations in the EnvZ/OmpR two-component regulatory system, including mutations in the outer membrane porin regulator (ompR) gene in strains EN01 and S7 and mutations in the environmental sensor kinase Z (envZ) gene in strains IT6 and S15 were detected. Similarly, mutations in genes coding for the different subunits of the DNA-directed RNA polymerase in strains IL9, S15, and DIA were observed. Lastly, mutations in genes coding for two proteins of the AcrABZ-TolC multidrug efflux pump in strains B, S5P8, S10, S12, S15, and MEX20 were observed. Taken together, benzoxazinoid resistance in *Photorhabdus* is a highly polygenic trait. The relative importance of the different mutations for benzoxazinoid resistance remains to be determined.

**Table 3:** Description of the different nonsynonymous gene mutations occurred during experimental evolution in MBOA-selected strains compared to LB-selected strains. Refer to Fig. 2 for graphical representations of the different mutations

Strain ID	Gene	Mutation type	Gene product name	Putative function
M-EN01	nhaB	SNP (c.1061C > T; p.Pro354Leu)	Na(+)/H(+) antiporter	Extrudes sodium in exchange for external protons
	ompR	SNP (c.614 T > G; p.Val205Gly)	Transcriptional regulatory protein	Osmoregulation and regulation of porins
	mppO	SNP (c.842G > T; p.Arg281Leu)	Enduracididine beta-hydroxylase	Oxidoreductase
M-IT6	envZ	SNP (c.1016G > A; p.Arg339Gln)	Osmolarity sensor protein	Osmoregulation and regulation of porins
M-IR2	fabR	SNP (c.116G > A; p.Arg39His)	HTH-type transcriptional regulator	Negative regulator of un-saturated fatty acid biosynthesis
M-IL9	rpoB	SNP (c.1655C > T; p.Pro552Leu)	DNA-directed RNA polymerase subunit beta	Transcription of DNA to RNA
M-TT01	waaA	SNP (c.668C > T; p.Thr223Ile)	3-deoxy-D-manno-octulosonic acid transferase	Lipopolysaccharide biosynthesis
	wzy	Stop gain (c.965G > A; p.Trp322*)	O-antigen polymerase	Polymerization of O-antigens
M-B	acrB	SNP (c.1390G > C; p.Gly464Arg)	Multidrug efflux pump subunit	Part of drug efflux protein complex
M-S5P8	acrB	SNP (c.1421 T > C; p.Ile474Thr)	Multidrug efflux pump subunit	
M-S7	ompR	SNP (c.659G > A; p.Arg220His)	Transcriptional regulatory protein	Osmoregulation and regulation of porins
M-S10	acrZ	SNP (c.7G > C; p.Glu3Gln)	Multidrug efflux pump accessory protein	Part of drug efflux protein complex
M-S12	acrB	SNP (c.1208G > A; p.Gly403Asp)	Multidrug efflux pump subunit	Part of drug efflux protein complex
M-S15	acrB	SNP (c.2999G > A; p.Gly1000Asp)	Multidrug efflux pump subunit	
	ychP	SNP (c.379A > G; p.Thr127Ala)	Inverse autotransporter beta domain-containing protein	Autotransporter
	panF	Frameshift (c.1167dupC; p.Glu390fs)	Sodium/pantothenate symporter	Pantothenate transmembrane transport
	envZ	Stop gain (c.373G > T; p.Glu125*)	Osmolarity sensor protein	Osmoregulation and regulation of porins
	rpoD	SNP (c.421G > A; p.Glu141Lys)	RNA polymerase sigma factor	Promotes the attachment of RNA polymerase
	xreA	SNP (c.52C > T; p.Arg18Cys)	XRE family transcriptional regulator	DNA-binding transcription factor
M-MEX20	acrB	SNP (c.2924G > A; p.Arg975His)	Multidrug efflux pump subunit	Part of drug efflux protein complex
M-DIA	rpoA	SNP (c.821C > A; p.Ala274Asp)	DNA-directed RNA polymerase subunit alpha	Transcription of DNA to RNA
	rpoC	SNP (c.3223C > T; p.Arg1075Cys)	DNA-directed RNA polymerase subunit beta	

### Benzoxazinoid resistance is associated with resistance to other xenobiotics

**Fig. 3:**
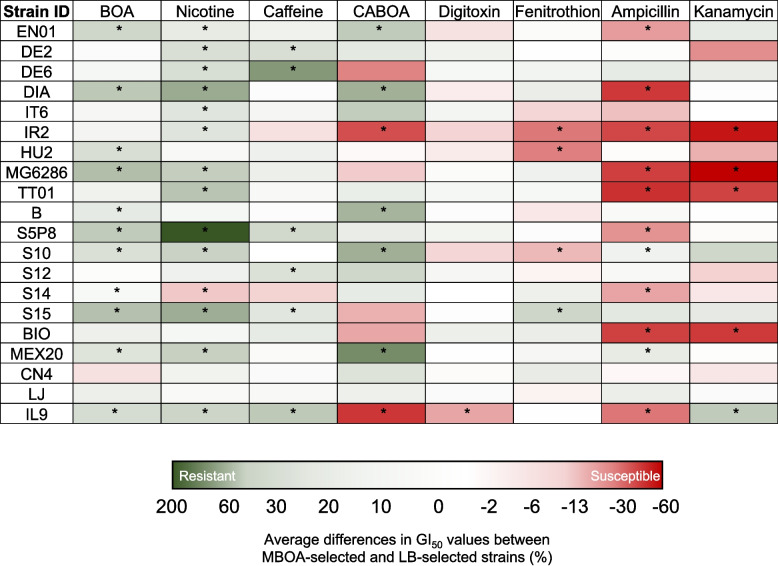
Experimental evolution in MBOA-containing culture medium alters bacterial resistance to multiple xenobiotics in a metabolite- and strain-specific manner. Heatmap on the average differences (%) in GI_50_ values of the bacterial strains selected in LB-medium (LB-selected, controls) and the GI_50_ values of the bacterial strains selected in MBOA-containing medium (MBOA-selected). Colour code indicate the magnitude and direction of the differences. Pink-Red colours indicate that MBOA-selected strains are more susceptible than LB-selected control strains, white colours that MBOA-selected strains and LB-selected control strains do not differ in their resistance degree, and light green-dark green colours indicate that MBOA-selected strains are more resistant than LB-selected control strains. Asterisks indicate significant differences in average differences in GI_50_ values between MBOA-selected and LB-selected strains (%) by two-way ANOVA with Holm’s multiple-comparisons tests (*: p < 0.05). Experiments were conducted three independent times with one replicate each time (n = 3). BOA: 2-benzoxazolinone; CABOA: 6-chloroacetyl-2-benzoxazolinone; MBOA: 6-methoxy-2-benzoxazolinone. Refer to Fig. S2 for details on the GI_50_ values used for the calculations

Several of the MBOA-selected strains were also found with altered resistance to multiple environmental xenobiotics (Fig. 3, Fig S2A-H). In general terms, most of the MBOA-selected strains were significantly more resistant to 2-benzoxazolinone (BOA), nicotine, caffeine, and 6-chloroacetyl-2-benzoxazolinone (CABOA) than their LB-selected counterparts (Fig. 3, Fig S2A-H). Little changes in their resistance to digitoxin were observed. However, most of the MBOA-selected strains were significantly more susceptible to fenitrothion, ampicillin, and kanamycin, apart from S15 that was more resistant to fenitrothion, MEX20 to ampicillin, and IL9 to kanamycin (Fig. 3, Fig S2A-H). Potential genetic regulatory elements can also be inferred from the genetic variant dataset (Table 3, Fig. S3). For instance, mutation in DNA-directed RNA polymerase often resulted in increased resistance to BOA and nicotine, and in decreased resistance to ampicillin (Fig. S3). The involvement of AcrABZ-TolC multidrug efflux pump in resistance to BOA and nicotine is also likely, as the resistance of most of the strains with mutations in its regulatory genes were more resistant to this compound (Fig. S3). Taken together, the evolution of resistance to benzoxazinoid MBOA results in cross-resistance and collateral sensitivity to other environmental xenobiotics.

### Costs of resistance to xenobiotics in Photorhabdus

To determine whether MBOA selection results in potential costs, we measured the growth of a subset of MBOA- and LB-selected strains in regular liquid medium. We selected the 20 strains that showed the highest increase in MBOA resistance. Across the different strains, negative, neutral, and positive effects in bacterial growth after the experimental evolution in MBOA were observed (Fig. S1). Most of the effects were significant, but minor, however, and only the growth of two strains, IL9 and HU2, was reduced by more than 20% (Fig. S1). Thus, the evolution of benzoxazinoid resistance can affect bacterial growth, but does not necessarily result in fitness-relevant costs under the given test conditions.

## Discussion

The western corn rootworm (WCR) is one of the most devastating insect pests of maize crops worldwide and causes extensive crop damage and consequent direct economic losses exceeding $2 billion US dollars annually (Wechsler and Smith 2018). A promising approach to control this pest is the use of entomopathogenic nematodes and their symbiotic bacteria [58, 59]. However, WCR has evolved plant toxin-mediated defensive mechanisms against these and several other natural enemies, and it is therefore partially resistant to their attack [15, 35, 36]. Here we used an experimental evolution approach to render entomopathogenic bacteria resistant to insect-sequestered plant toxins, and used this approach to better understand the prevalence, mechanisms, and side-effects of toxin resistance.

Experimental evolution coupled with genome-wide single-nucleotide polymorphism and structural variant analyses revealed the potential genetic bases and the polygenic nature of benzoxazinoid resistance in *Photorhabdus*. Mutations that impact different cellular processes including transmembrane transport, oxidoreductase activity, osmoregulation, membrane fatty acid, lipopolysaccharide and o-antigen biosynthesis, and DNA transcription, were observed after benzoxazinoid resistance evolution. In particular, genetic alterations in genes coding for the EnvZ/OmpR two-component regulatory system, coding for the different subunits of the DNA-directed RNA polymerase, and coding for proteins of the AcrABZ-TolC multidrug efflux pump were common across the different benzoxazinoid resistant strains. There are four primary mechanisms of antibiotic resistance in bacteria: i) modification of cellular targets to reduce antibiotic binding, ii) decreased cellular uptake of the antibiotic, iii) active expulsion of the antibiotic via enhanced or modified efflux pumps, and iv) enzymatic inactivation of the antibiotic [52]. Our results show that benzoxazinoid resistance can be achieved by similar mechanisms, apart from enzymatic inactivation of the antibiotic, which is unlikely to result after experimental evolution, although several bacteria can enzymatically metabolize benzoxazinoids [53, 54]. The evolution of resistance to one antibiotic or drug can result in increased resistance to another antibiotic, phenomenon known as cross-resistance [55–57]. Conversely, the evolution of resistance to one antibiotic or drug can also result in decreased resistance to another, a phenomenon known as collateral sensitivity [56]. We observed that benzoxazinoid resistance evolution often resulted in cross-resistance to 2-benzoxazolinone (BOA), 6-chloroacetyl-2-benzoxazolinone (CABOA), nicotine, and/or caffeine, and in collateral sensitivity to ampicillin and/or kanamycin. In addition, evolution of resistance is often constrained by physiological or ecological trade-offs. For example, resistance to xenobiotics is often associated with different types of costs in bacteria, including reduced growth, impaired competitive performance and metabolic imbalance [58, 59]. Contrary to our expectations, we observed little impact of evolving benzoxazinoid resistance on bacterial growth. Only two strains suffered severe growth penalties after experimental evolution. Some of the cellular processes impacted by the observed mutations can cause growth abnormalities. Bacterial mutants defective in lipopolysaccharide biosynthesis often exhibit aberrant growth [15, 60]. Similarly, mutations in the different subunits of the DNA-directed RNA polymerase cause also reduced bacterial growth [61–66]. Hence, our study uncovers specific mutations that can likely increase resistance to environmental xenobiotics with minimal growth penalties, allowing to maximize the benefits of targeted genetic engineering of these biological control agents. Future experiments will aim at understanding the relative importance of the individual mutations through targeted genetic engineering.

Experimental evolution coupled with genome-wide single-nucleotide polymorphism and structural variant analyses revealed the potential genetic bases and the polygenic nature of benzoxazinoid resistance in *Photorhabdus*. Mutations that impact different cellular processes including transmembrane transport, oxidoreductase activity, osmoregulation, membrane fatty acid, lipopolysaccharide and o-antigen biosynthesis, and DNA transcription, were observed after benzoxazinoid resistance evolution. In particular, genetic alterations in genes coding for the EnvZ/OmpR two-component regulatory system, coding for the different subunits of the DNA-directed RNA polymerase, and coding for proteins of the AcrABZ-TolC multidrug efflux pump were common across the different benzoxazinoid resistant strains. There are four primary mechanisms of antibiotic resistance in bacteria: i) modification of cellular targets to reduce antibiotic binding, ii) decreased cellular uptake of the antibiotic, iii) active expulsion of the antibiotic via enhanced or modified efflux pumps, and iv) enzymatic inactivation of the antibiotic [60]. Our results show that benzoxazinoid resistance can be achieved by similar mechanisms, apart from enzymatic inactivation of the antibiotic, which is unlikely to result after experimental evolution, although several bacteria can enzymatically metabolize benzoxazinoids [61, 62]. The evolution of resistance to one antibiotic or drug can result in increased resistance to another antibiotic, phenomenon known as cross-resistance [63–65]. Conversely, the evolution of resistance to one antibiotic or drug can also result in decreased resistance to another, a phenomenon known as collateral sensitivity [64]. We observed that benzoxazinoid resistance evolution often resulted in cross-resistance to 2-benzoxazolinone (BOA), 6-chloroacetyl-2-benzoxazolinone (CABOA), nicotine, and/or caffeine, and in collateral sensitivity to ampicillin and/or kanamycin. In addition, evolution of resistance is often constrained by physiological or ecological trade-offs. For example, resistance to xenobiotics is often associated with different types of costs in bacteria, including reduced growth, impaired competitive performance and metabolic imbalance [66, 67]. Contrary to our expectations, we observed little impact of evolving benzoxazinoid resistance on bacterial growth. Only two strains suffered severe growth penalties after experimental evolution. Some of the cellular processes impacted by the observed mutations can cause growth abnormalities. Bacterial mutants defective in lipopolysaccharide biosynthesis often exhibit aberrant growth [15, 68]. Similarly, mutations in the different subunits of the DNA-directed RNA polymerase cause also reduced bacterial growth [69–74]. Hence, our study uncovers specific mutations that can increase resistance to environmental xenobiotics with minimal growth penalties, allowing to maximize the benefits of targeted genetic engineering of these biological control agents.

Although we identified multiple mutations across different *Photorhabdus* strains that correlate with resistance to 6-methoxy-2-benzoxazolinone (MBOA), the relative contribution of each mutation to the resistant phenotype remains unresolved. Resistance mechanisms in bacteria often arise through a combination of genetic alterations, including point mutations, insertions/deletions, and regulatory changes, which can act synergistically or independently [75, 76]. Given the polygenic nature of resistance, deciphering the functional relevance of each mutation requires systematic dissection through targeted mutagenesis, such as site-directed mutagenesis and allelic exchange to individually introduce and/or revert the candidate mutations in an isogenic background. This strategy will help determine whether specific mutations are necessary and/or sufficient for MBOA resistance, or whether they act in combination with other genomic changes. Similar approaches have been successfully used to parse the contributions of individual mutations in antimicrobial resistance evolution, and are essential for establishing causal relationships [15, 77]. By functionally validating the observed mutations, we aim to build a clearer understanding of the molecular mechanisms underlying MBOA resistance in *Photorhabdus*, and to distinguish between adaptive, compensatory, and neutral mutations.

## Conclusion

Our study provides deeper insight into the genes and types of mutations that can be engineered in *Photorhabdus* bacteria to increase their biological control potential against an agricultural pest of global relevance, but that will have minimal undesired cross-resistance, collateral sensitivity, and growth effects. Our approach also provides molecular tools to develop better biological agents, which are essential for more sustainable and ecologically friendly agricultural practices.

## Supplementary Information

Supplementary material 1.

Supplementary material 2.

Supplementary material 3.

Supplementary material 4.

Supplementary material 5.

## Data Availability

Gene sequences of ancestral, LB-, and MBOA-selected strains are available as a supplementary material (Data S1). An excel file containing all the datasets generated and/or analysed during the current study are available as a supplementary material (Data S2).
