# Supplementary material for: Genetic architecture of resistance to plant secondary metabolites in Photorhabdus entomopathogenic bacteria
Source: BMC Genomics. 2025 Oct 30;26:975. doi: 10.1186/s12864-025-12067-x (PMC12577137; doi:10.1186/s12864-025-12067-x)
Supplement: Supplementary file 1 — Supplementary material 1. [file 12864_2025_12067_MOESM1_ESM.pdf]

A)

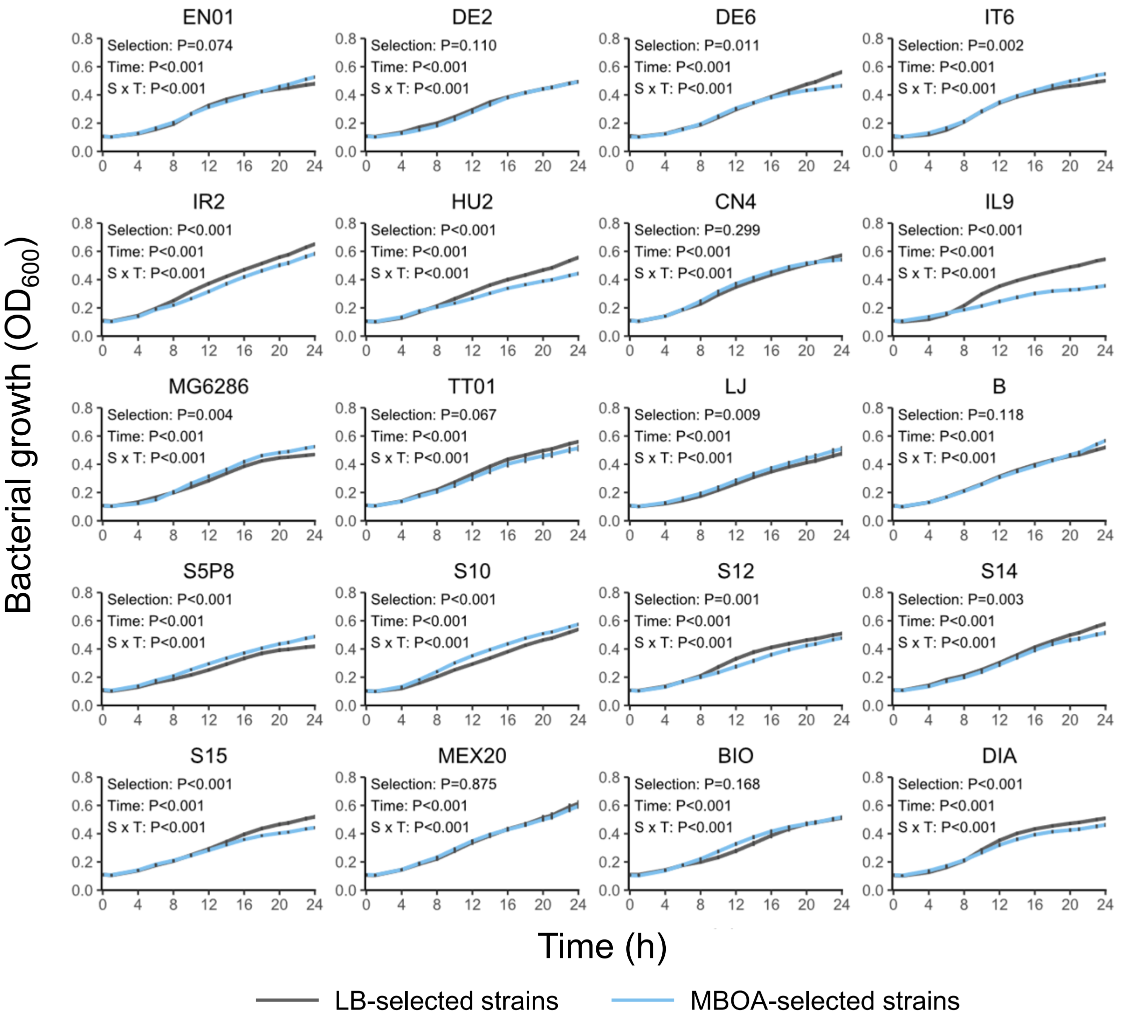

B)

Strain ID	Δ Growth (%)
IL9	-35
HU2	-20
DE6	-17
S15	-15
S14	-11
IR2	-11
DIA	-9
TT01	-8
S12	-6
CN4	-6
MEX20	-3
DE2	0
BIO	1
S10	7
LJ	7
B	9
IT6	10
EN01	10
MG6286	12
S5P8	17

Figure S1. *Selection on MBOA impacts bacterial growth in a strain-specific manner.* A) Mean (±SEM) bacterial growth in liquid LB during 24h. Bacterial growth was estimated by measuring optical densities of liquid cultures at 600nm (OD_{600}). Grey lines represent bacterial growth of LB-selected strains. Blue lines represent bacterial growth of MBOA-selected strains. Differences between selection regime and timepoints were determined using two-way repeated measurements ANOVA with Holm's multiple comparisons test. B) Difference (%) in total growth of MBOA-selected strains and LB-selected strains after 24h. Positive values indicate that MBOA-selected strains grew more than LB-selected strains. Conversely, negative values indicate that MBOA-selected strains grew less than LB-selected strains. Experiments were conducted eight independent times with one replicate each time (n=8). MBOA: 6-methoxy-2-benzoxazolinone.
