# Supplementary material for: Genetic architecture of resistance to plant secondary metabolites in Photorhabdus entomopathogenic bacteria
Source: BMC Genomics. 2025 Oct 30;26:975. doi: 10.1186/s12864-025-12067-x (PMC12577137; doi:10.1186/s12864-025-12067-x)
Supplement: Supplementary file 2 — Supplementary material 2. [file 12864_2025_12067_MOESM2_ESM.pdf]

A)

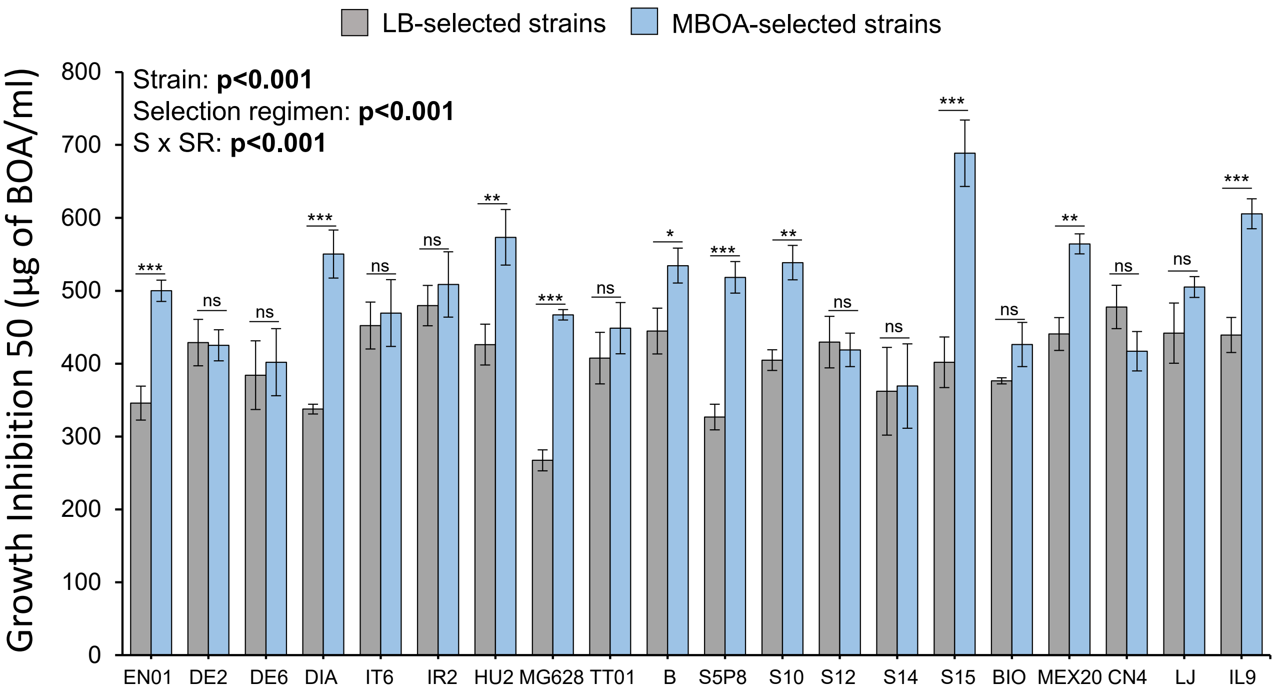

B)

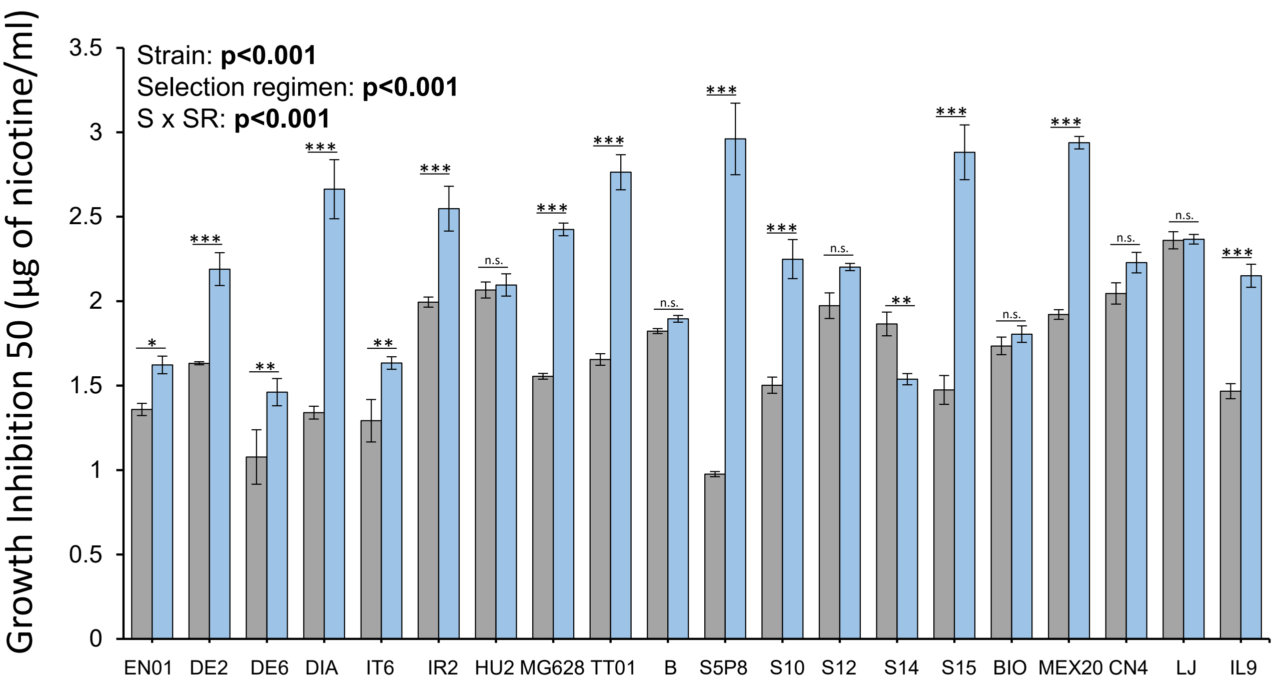

Continues below…

…Starts above

C)

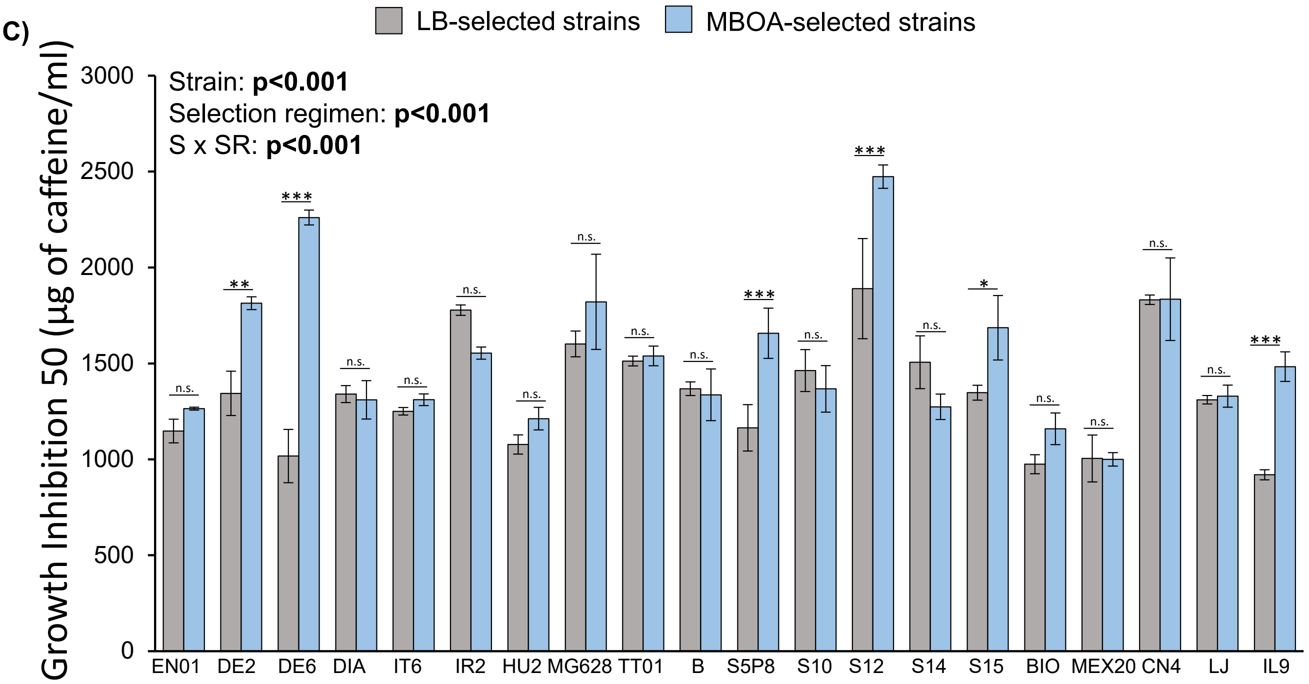

D)

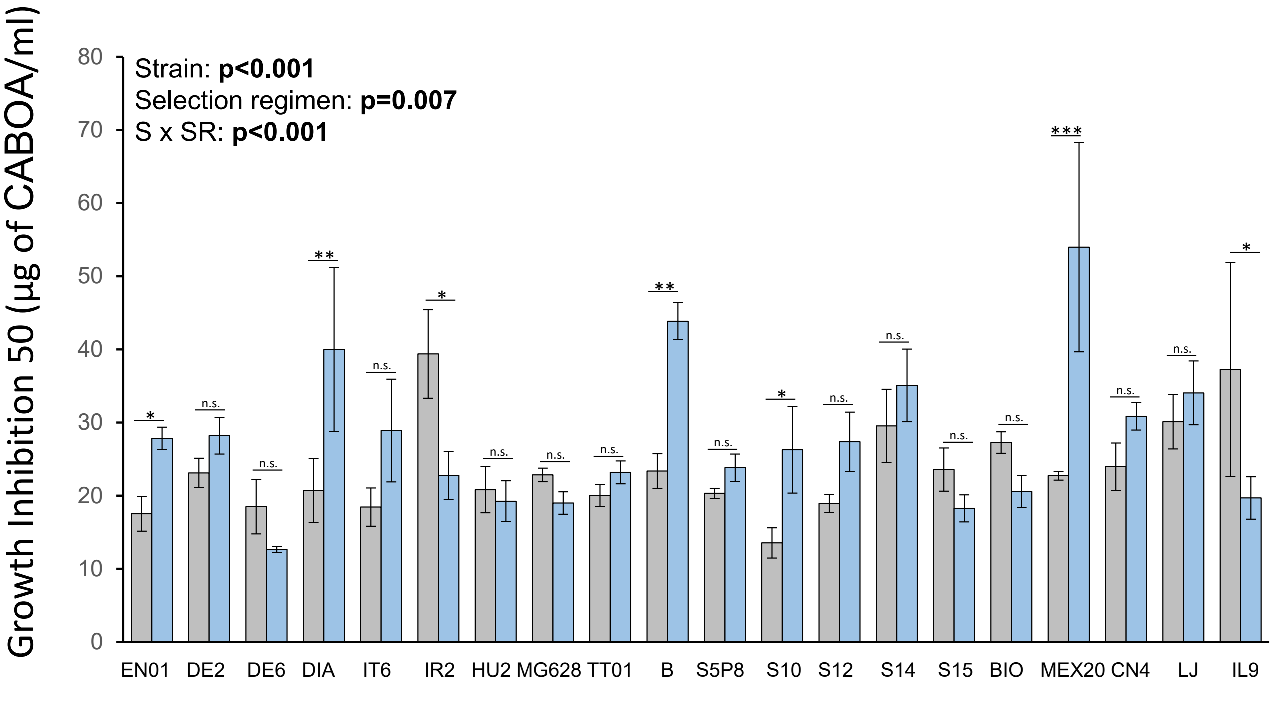

Continues below…

…Starts above

E)

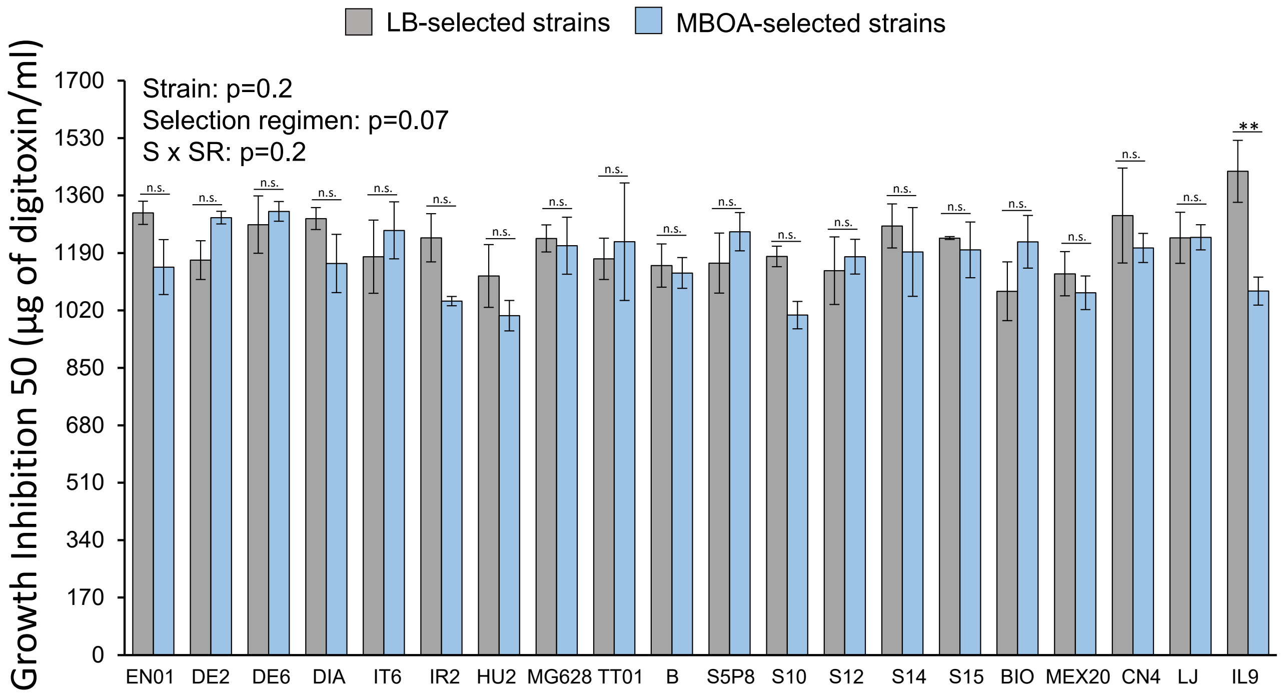

F)

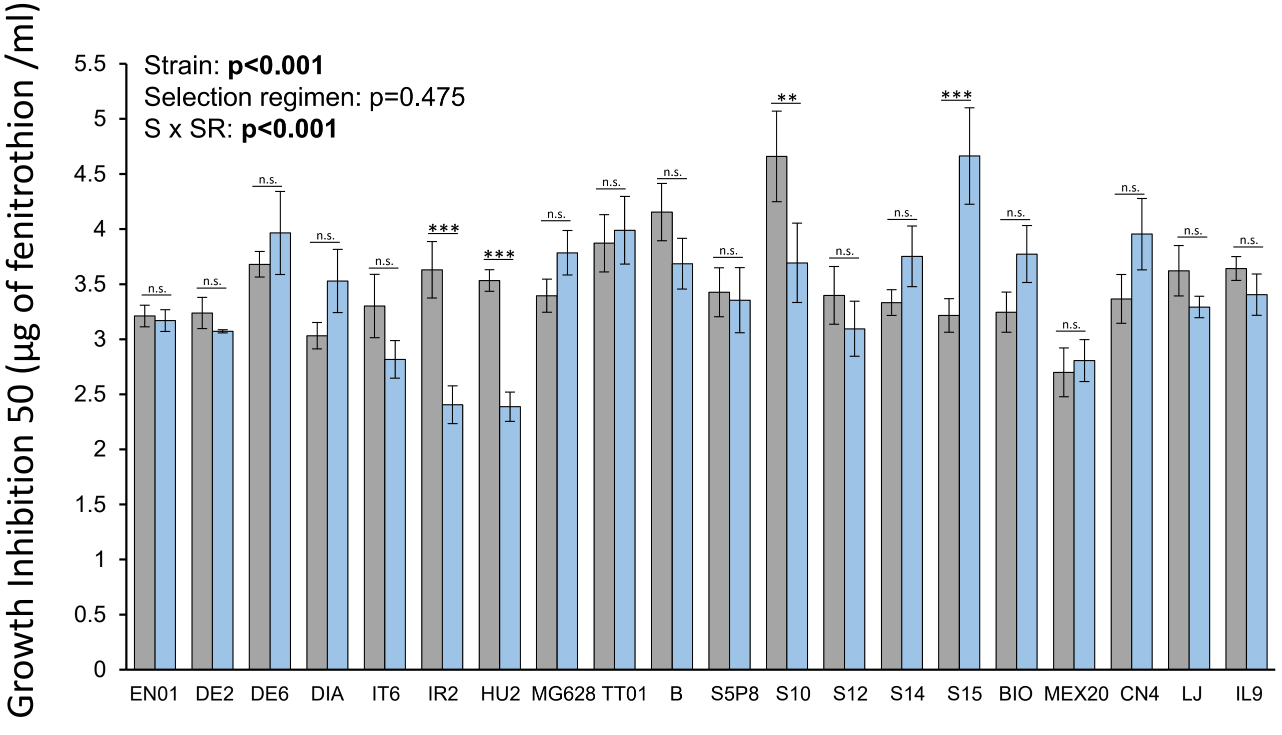

Continues below…

…Starts above

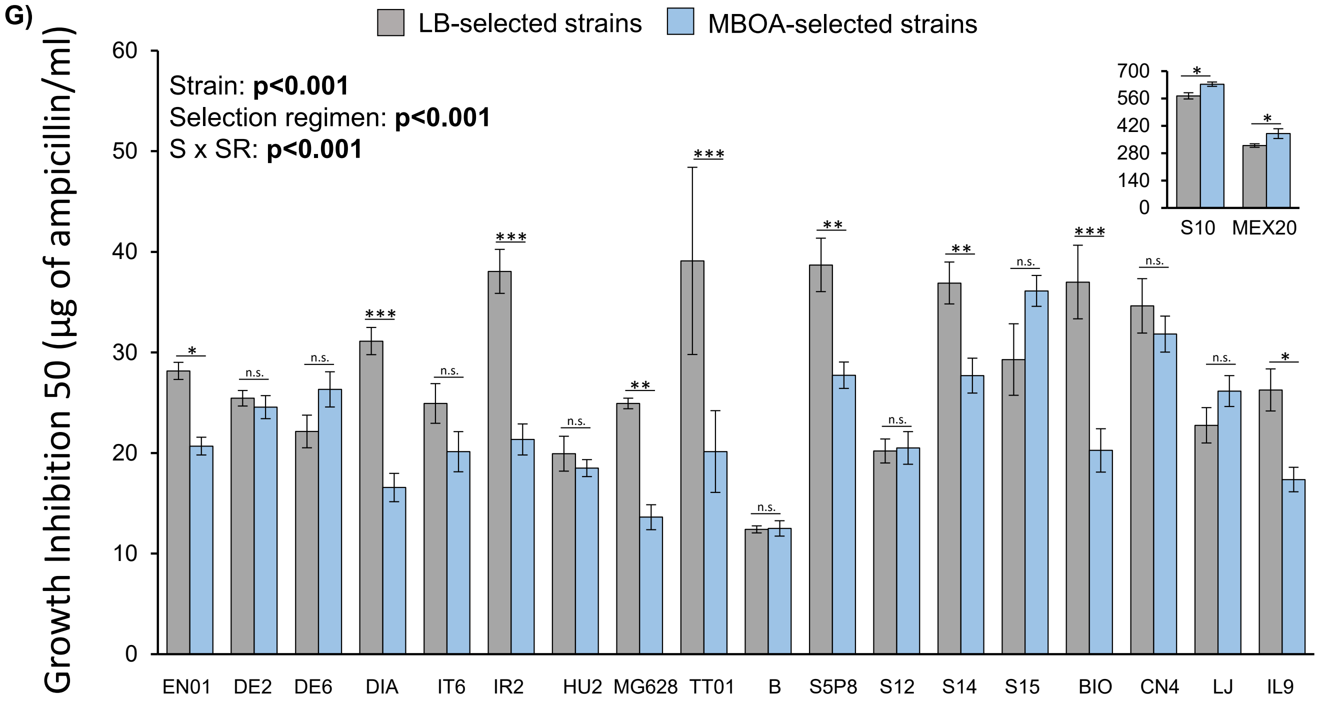

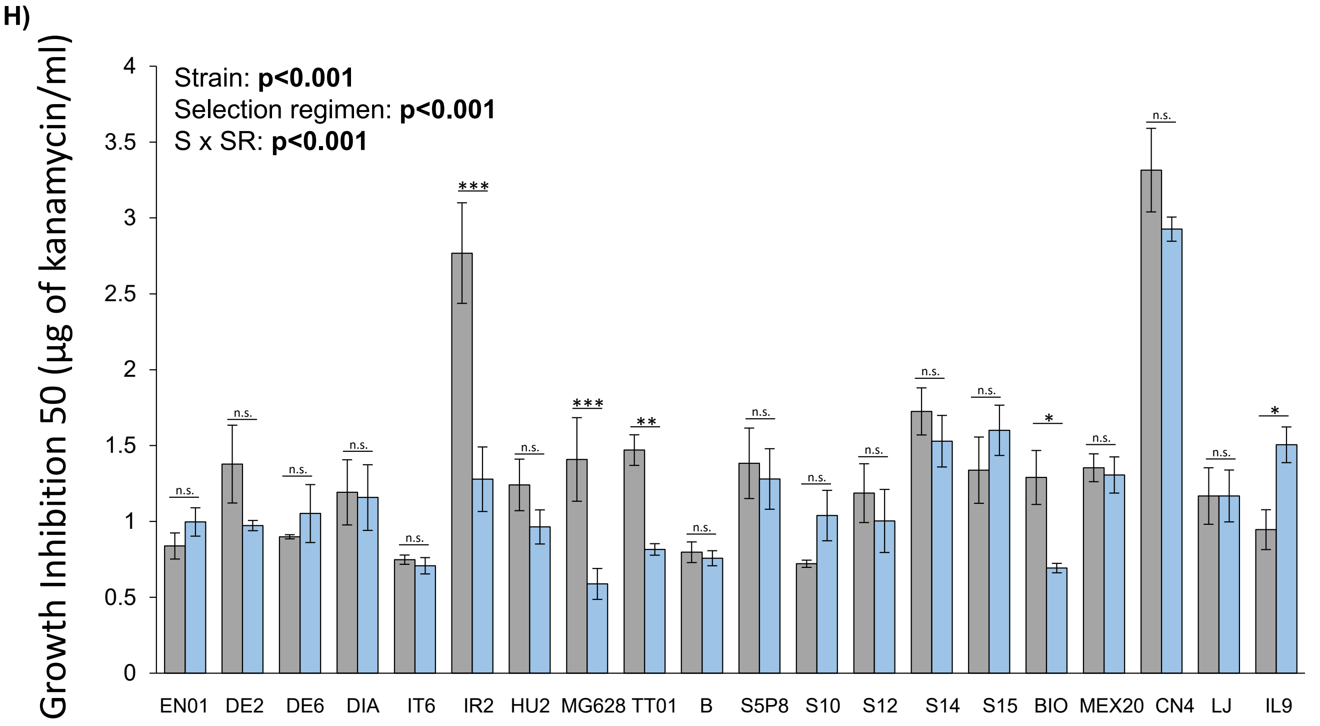

Figure S2. *Experimental evolution in MBOA-containing culture medium alters bacterial resistance to multiple xenobiotics in a metabolite- and strain-specific manner.* A-H) Mean (±SEM) metabolite concentration that inhibit bacterial growth by 50% (Growth Inhibition 50, GI_{50}) in the bacterial strains selected in LB-medium (LB-selected, grey bars) and in the bacterial strains selected in MBOA-containing medium (MBOA-selected, blue bars). The following metabolites were evaluated: A) 2-benzoxazolinone (BOA), B) Nicotine, C) Caffeine, D) 6-chloroacetyl-2-benzoxazolinone (CABOA), E) Digitoxin, F) fenitrothion, G) Ampicillin, and H) kanamycin. Asterisks indicate significant differences in GI_{50} values between LB-selected and MBOA-selected strains by two-way ANOVA with Holm's multiple-comparisons tests (*: p<0.05, **: p<0.01, ***: p<0.001). Experiments were conducted three independent times with one replicate each time (n=3). Refer to Table S1 for details on the concentration ranges tested and biological activities of the different metabolites evaluated. MBOA: 6-methoxy-2-benzoxazolinone.
