# Supplementary material for: Genetic architecture of resistance to plant secondary metabolites in Photorhabdus entomopathogenic bacteria
Source: BMC Genomics. 2025 Oct 30;26:975. doi: 10.1186/s12864-025-12067-x (PMC12577137; doi:10.1186/s12864-025-12067-x)
Supplement: Supplementary file 3 — Supplementary material 3. [file 12864_2025_12067_MOESM3_ESM.pdf]

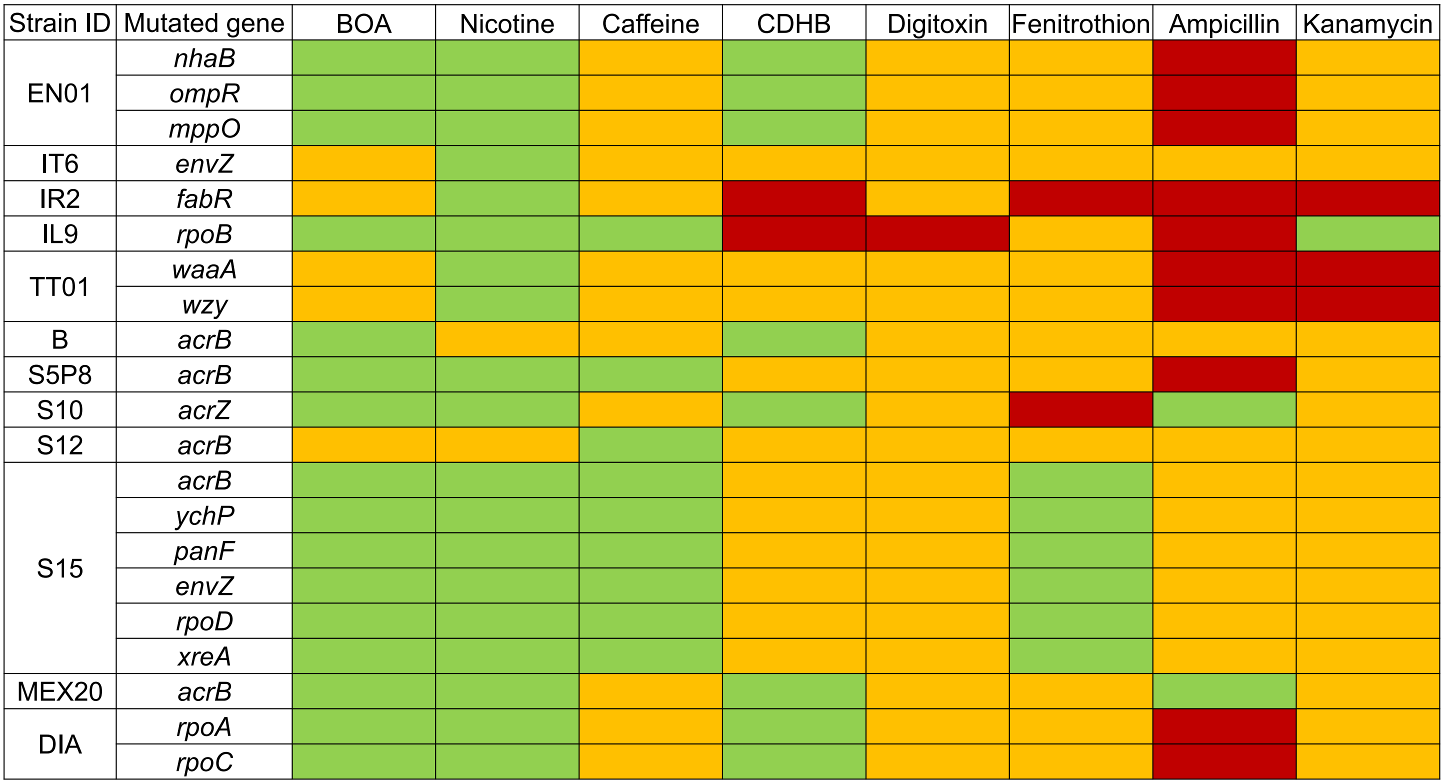

Figure S3. *Experimental evolution in MBOA-containing culture medium alters bacterial resistance to multiple xenobiotics in a metabolite- and strain-specific manner.* Categorical heatmap of the resistance of MBOA-selected strains to different toxic metabolites compared to the resistance of LB-selected strains. Green, orange, and red colours indicate that the resistance degree significantly increased, did not change, or decrease, respectively, in MBOA-selected strains. Refer to Fig. 3 and S2 for details and quantitative data. MBOA: 6-methoxy-2-benzoxazolinone.
